# Supplementary material for: Association between different types of mass media and antenatal care visits in India: a cross-sectional study from the National Family Health Survey (2015–2016)
Source: BMJ Open. 2020 Dec 15;10(12):e042839. doi: 10.1136/bmjopen-2020-042839 (PMC7745528; doi:10.1136/bmjopen-2020-042839)
Supplement: Author's manuscript [file bmjopen-2020-042839.draft_revisions.pdf]

BMJ Open is committed to open peer review. As part of this commitment we make the peer review history of every article we publish publicly available.

When an article is published we post the peer reviewers' comments and the authors' responses online. We also post the versions of the paper that were used during peer review. These are the versions that the peer review comments apply to.

The versions of the paper that follow are the versions that were submitted during the peer review process. They are not the versions of record or the final published versions. They should not be cited or distributed as the published version of this manuscript.

BMJ Open is an open access journal and the full, final, typeset and author-corrected version of record of the manuscript is available on our site with no access controls, subscription charges or pay-per-view fees (http://bmjopen.bmj.com).

If you have any questions on BMJ Open's open peer review process please email info.bmjopen@bmj.com

BMJ Open

How can mass media help improve maternal and child health in India?

Journal:	*BMJ Open*
Manuscript ID	bmjopen-2020-042839
Article Type:	Original research
Date Submitted by the Author:	16-Jul-2020
Complete List of Authors:	Dhawan, Dhriti; Dana-Farber Cancer Institute, Medical Oncology<br>Pinnamaneni, Ramya; Harvard University T H Chan School of Public Health,<br>Bekalu, Mesfin; Harvard University T H Chan School of Public Health,<br>Viswanath, Kasisomayajula; Harvard University T H Chan School of Public Health, ; Dana-Farber Cancer Institute, Medical Oncology
Keywords:	EPIDEMIOLOGY, OBSTETRICS, PUBLIC HEALTH, REPRODUCTIVE MEDICINE

SCHOLARONE™
Manuscripts

I, the Submitting Author has the right to grant and does grant on behalf of all authors of the Work (as defined in the below author licence), an exclusive licence and/or a non-exclusive licence for contributions from authors who are: i) UK Crown employees; ii) where BMJ has agreed a CC-BY licence shall apply, and/or iii) in accordance with the terms applicable for US Federal Government officers or employees acting as part of their official duties; on a worldwide, perpetual, irrevocable, royalty-free basis to BMJ Publishing Group Ltd ("BMJ") its licensees and where the relevant Journal is co-owned by BMJ to the co-owners of the Journal, to publish the Work in this journal and any other BMJ products and to exploit all rights, as set out in our licence.

The Submitting Author accepts and understands that any supply made under these terms is made by BMJ to the Submitting Author unless you are acting as an employee on behalf of your employer or a postgraduate student of an affiliated institution which is paying any applicable article publishing charge ("APC") for Open Access articles. Where the Submitting Author wishes to make the Work available on an Open Access basis (and intends to pay the relevant APC), the terms of reuse of such Open Access shall be governed by a Creative Commons licence – details of these licences and which Creative Commons licence will apply to this Work are set out in our licence referred to above.

Other than as permitted in any relevant BMJ Author's Self Archiving Policies, I confirm this Work has not been accepted for publication elsewhere, is not being considered for publication elsewhere and does not duplicate material already published. I confirm all authors consent to publication of this Work and authorise the granting of this licence.

For peer review only

How can mass media help improve maternal and child health in India?

Correspondence to:

Dhriti Dhawan, PhD, MPH;

Dana-Farber Cancer Institute

450 Brookline Avenue, LW 601

Boston, MA 02215-5450
Tel: (617) 632-6586
Fax: (617) 582-8728

dhriti_dhawan@dfci.harvard.edu

Co-Authors:

1. **Dhriti Dhawan, PhD, MPH;**

   Department: Medical Oncology

   Institution: Dana-Farber Cancer Institute, Boston, MA, USA,

   Full address: 450 Brookline Ave. Boston, MA 02215

2. **Ramya Pinnamaneni, MBBS, MPH;**

   Department: Social and Behavioral Sciences

   Institution: Harvard T.H Chan School of Public Health, Boston, MA, USA,

   Full address: 450 Brookline Ave. Boston, MA 02215

3. **Mesfin Awoke Bekalu, PhD;**

   Department: Social and Behavioral Sciences

   Institution: Harvard T.H Chan School of Public Health, Boston, MA, USA,

   Full address: 450 Brookline Ave. Boston, MA 02215

4. **K. Viswanath, PhD**

   Department: Social and Behavioral Sciences

   Institution: Harvard T.H Chan School of Public Health, Boston, MA, USA,

   Full address: 450 Brookline Ave. Boston, MA 02215

Word count: 3403

For peer review only

Abstract

Objective To generate evidence for the potential role of different types of media in creating awareness about obtaining adequate Ante Natal Care (ANC) in Indian women.

Design A cross-sectional study design, analyzing data from India's National Family Health Survey 4 (2015-16) (NFHS-4).

Setting Urban and rural India.

Participants From the NFHS-4, women who had given birth in the last five years before survey administration were included in this study. Women with missing information about the number of ANC visits and their caste were excluded from the study. 187,894 women were included in the final analytical sample.

Primary outcome measures Logistic regression analysis was conducted to determine the association between ANC utilization with mass media use.

Results As per WHO's recommendation prior to 2016, 51.7% of pregnant women in India had at least four ANC visits. However, as per WHO's revised recommendation in 2016, only 20.3% of pregnant women had at least eight ANC visits. In our study, we observed that high exposure to all four types of mass media was positively associated with making at least eight ANC visits, in both rural and urban areas. Compared to other media, women who watched television almost every day had the highest odds of making at least eight ANC visits, especially in rural areas.

Conclusions Our findings emphasize the need for increased awareness about obtaining adequate ANC visits in India, to improve maternal, neonatal, and child health outcomes. Our study highlights that television penetration is broader than other forms of media and has the potential to create awareness about health in both urban and rural populations. These findings can inform ANC related health awareness campaigns in the country to allocate resources to the appropriate media sources to encourage healthy behaviors.

Keywords Antenatal care, Maternal health, Child health, Health communication, LMIC

Word count 3406

For peer review only

For peer review only - http://bmjopen.bmj.com/site/about/guidelines.xhtml

Article Summary

Strengths and limitations of this study

- Our results are derived from a nationally representative survey and are generalizable to women in India (strength).
- This data enables us to determine trends stratified by urbanicity, states, SES, caste, and religion, which can further help to narrow down the at-risk populations and design tailored interventions (strength).
- The cross-sectional design of the data limits the ability to derive any causal relationships between the variables. Nonetheless, the strong association between media use and ANC visits suggest the potential promise of media in maternal health promotion (limitation).
- The dataset has no information about the type of ANC-related media content available to women in India (limitation).
- Majority of the NFHS-4 data is self-reported, making it prone to recall bias and under- or over-reporting of behaviors (limitation).

For peer review only

For peer review only - http://bmjopen.bmj.com/site/about/guidelines.xhtml

Introduction

Ante Natal care (ANC) is widely acknowledged as a critical tool in reducing stillbirths, complications in pregnancy, and promoting positive pregnancy experience.[1] Components of ANC are risk identification, prevention and management of pregnancy-related or concurrent diseases, and maternal health education and health promotion.[1] ANC is conducive for a healthy pregnancy and improves the chances of survival for the mother as well as for the child. It has also been shown to support the growth and development of the child until the age of five years.[2] Women who utilize ANC services are more likely to use other essential services such as counseling for breastfeeding, nutrition, postpartum family planning as well as childhood immunization.[3]

Before 2016, the World Health Organization (WHO) recommended at least four ANC visits during normal pregnancy and that the first visit be completed within the first 8-12 weeks of conception.[1] However, in 2016, the WHO changed its recommendation to a minimum of eight ANC visits.[1] This was because, compared to four visits, eight or more ANC visits have the potential to reduce perinatal deaths by up to 8 per 1000 births, as it provides more opportunities for detecting and treating potential complications.[4] Despite these recommendations, women of low- and middle-income countries have low ANC coverage.[1,5] According to UNICEF, there are wide disparities in ANC coverage across countries with the lowest levels in sub-Saharan Africa and South Asia.[6] There is an urgent need to create awareness about the benefits of utilizing ANC services and support interventions aimed towards increasing their utilization to improve maternal, neonatal as well as child health. We need strategies that can promote ANC service utilization at scale.

Mass media campaigns

Mass media are critical tools in promoting health through two key strategies: one, their widespread penetration promotes broad reach to key audiences across boundaries, and two, exposure to specific messages in the media are known to shape public knowledge, attitudes, beliefs and behaviors.[7] Routine use of media can influence both pro- and anti-healthy behaviors.[8] Media campaigns have been extensively used over the past few

For peer review only - http://bmjopen.bmj.com/site/about/guidelines.xhtml

decades to induce behavioral changes in populations, especially in the context of substance use, such as tobacco, alcohol, and illicit drugs.

Creating awareness through mass media can encourage positive or discourage negative health-related behaviors across populations, through direct and indirect pathways.[8] These behavioral changes occur passively as a result of routine media use.[9] Mass media campaigns can invoke cognitive or emotional responses at an individual level by informing the population about the benefits and risks of a particular health-related behavior. These campaigns frequently associate emotions with achieving change, strengthening the probability of alteration, and increasing the likelihood of adopting new behaviors.[10] Media campaigns have created awareness about health-related issues such as prevention of heart disease, cancer screening and prevention, sex-related behaviors, and child survival amongst others.[9] Many low-income countries face poor child survival before five years of age because of inadequate treatment of diarrhea, non-vaccination for preventable diseases, inadequate ANC, and inadequate breastfeeding.[11,12] Mass media campaigns have been used to spread awareness and educate populations about each of these causes, and have resulted in mixed evidence for success.[9]

For creating awareness amongst large audiences, the most popular modes of media are television and radio. However, print media, such as magazines and newspapers, and outdoor media, such as billboards and posters, have also proven effective.[9] Broadcasting important messages related to ANC through mass media may help in increasing awareness at the population level.[2]

Antenatal Care in India

Our study examines the association between media use and ANC visits in India. Indian women carry a heavy burden of poor nutritional status and infections, which is associated with high child mortality as well as maternal mortality.[13] In 2017, India recorded 35,000 maternal deaths- second highest in the world.[14] In 2019, India recorded 882,000 children under-five deaths and 549,000 neonatal deaths- highest in the world.[15] ANC

For peer review only - http://bmjopen.bmj.com/site/about/guidelines.xhtml

visits are of high importance in India because they can help prevent adverse effects on the health of women and children, and reduce the risk of under-five and neonatal deaths.[12]

Many intervention programs in India use mass media as one of the communication tools to create health-related awareness.[16,17] For example, the National Nutrition Mission of India (*POSHAN Abhiyaan*) plans to utilize various platforms including mass media to inspire a people's movement, *Jan Andolan*, by creating awareness about nutrition and ensuring the engagement of the population at different levels.[18] The objective of our study is to generate evidence for the potential role of different types of media in creating awareness about obtaining adequate ANC. Specifically, within ANC, we tested the association of the self-reported number of ANC visits with the frequency of use of four types of mass media: newspapers/magazines, radio, television, and movies.

Methods

Data source

This analysis utilizes individual-level data from India's National Family Health Survey 4 (NFHS-4), 2015-16.[19] The NFHS series are nationally representative and cross-sectional surveys that provide data on a range of demographic, socioeconomic, maternal and child health outcomes, reproductive health, and family planning. NFHS-4 gathered information from 601,509 households, 699,686 women, and 112,122 men, with a response rate of 97%. Of the women, 190,898 women had given birth in the last five years. 1,854 women were missing information about their number of ANC visits during pregnancy, and 1,150 women were missing information about their caste. These women were excluded from the study, thereby leaving 187,894 women in the analytical sample.

Sample design

The NFHS-4 used a two-stage stratified sampling design. The 2011 census served as the sampling frame for the selection of primary sampling units (PSUs). The samples were stratified by rural and urban areas, and villages were PSUs in the rural areas, and census

For peer review only - http://bmjopen.bmj.com/site/about/guidelines.xhtml

enumeration blocks were PSUs in the urban areas. Before the main survey, for each PSU, a household mapping and listing operation were carried out. Selected PSUs with at least 300 households were further segmented into 100-150 household segments. Systematic sampling was used to randomly select two of the segments, with probability proportional to segment size. Hence, an NFHS-4 cluster consists of either a PSU or a segment of a PSU. Twenty-two households were randomly selected with systematic sampling in every selected rural and urban cluster.

Outcome measure

For their most recent pregnancy in the last five years, women were asked: “How many times did you receive antenatal care during this pregnancy?”. This was a continuous variable, which was recoded to a binary variable as women who had eight or more antenatal care visits and women who did not.

Exposures

Women were asked “Do you read a newspaper or magazine almost every day, at least once a week, less than once a week or not at all?”, “Do you listen to the radio almost every day, at least once a week, less than once a week or not at all?”, and “Do you watch television almost every day, at least once a week, less than once a week or not at all?”. These were categorical variables with the four given option categories. These variables were recoded to binary variables with response options as “Almost every day” and “Not every day” (combining the responses at least once a week, less than once a week and not at all). They were also asked, “Do you usually go to a cinema hall or theatre to see a movie at least once a month?” with response options “Yes” and “No”.

Covariates

The age of the participants was categorized in increments of five years and ranged from 15 to 49 years of age. Educational attainment of the participants was categorized per the Indian educational system: No education, Incomplete primary education, Complete primary education, Incomplete secondary education, Complete secondary education, and

For peer review only - http://bmjopen.bmj.com/site/about/guidelines.xhtml

Higher. Participants were grouped according to their marital status: Never married, Married, Widowed, Divorced, and Separated. In India, wealth has been established as a valid measure of socioeconomic status (SES). It can be defined as a measure of housing characteristics and possession of materials. Using principal component analysis, a wealth score was assigned to each participating household. Each person in the household was assigned a household score and ranked according to it. The distribution was divided into quintiles. Wealth of the participants was categorized accordingly.[19] The Caste system in India has contributed to the social marginalization of people who belong to certain groups and may potentially influence access to healthcare including maternal healthcare.[20] NFHS reports four categories: Scheduled Castes, Scheduled Tribes, Other Backward Classes, and General. The urbanicity of the participants was categorized as rural or urban. The religion of the participants was categorized as Hindu, Muslim, Christian, Sikh, and other.

Statistical analysis

Frequencies of key outcome and exposure variables as well as the covariates were obtained to describe the study sample. Participants with "Do not know" responses for our variables of interest were recoded to missing and excluded from the present study. Logistic regression analysis was conducted to determine the association between our outcome variable: "At least eight antenatal care visits", and our respective exposure variables: "Newspaper/Magazine use", "Radio use", "Television use", or "Movie use". Multivariable logistic regression was conducted with each of the media use variables while adjusting for other media use variables as well as the covariates. The data were analyzed and weighted using R version 3.6.1 (2019-07-05). Survey weights and clustering within PSUs were included in the analysis and the survey-weighted logistic regression was carried with the R survey package.

Results

Based on our analysis of the NFHS-4 survey, Indian women reported making an average of 4.8 ANC visits (SE ±0.03) during pregnancy. 51.7% of pregnant women in India

For peer review only - http://bmjopen.bmj.com/site/about/guidelines.xhtml

received at least four ANC visits, as was recommended by WHO before 2016. However, as per WHO's revised recommendation in 2016, only 20.3% of pregnant Indian women received at least eight ANC visits. 9.9%, 3.3%, and 55.1% of our target population use newspaper/magazine, radio, and television, respectively, almost every day, and 7.4% of our target population go to the cinema to watch a movie at least once a month (Table 1). Needless to say, television penetration and reach are broad compared to other media. The percentage of women making at least eight ANC visits increased with an increase in the indicators of SES such as education attainment after complete primary education, and wealth. Comparing media use characteristics amongst rural and urban women, each type of media use was more prevalent in an urban context. The sample size in each cell fulfilled the conditions to permit the use of multivariable logistic regressions (Table 2).

Media and at least eight ANC visits

After adjusting for age, educational attainment, current marital status, wealth, caste, urbanicity, religion, and other media use variables, women who used newspaper/magazine [OR 1.26, 95%CI 1.18-1.35], used the radio [OR 1.29, 95% CI 1.15-1.44], used television [OR 1.84, 95%CI 1.74-1.95] almost every day, or went to the cinema to see a movie at least once a month [OR 1.29, 95%CI 1.19-1.4] were more likely to receive at least eight ANC visits as compared to those who did not (Table 3).

Upon stratification of the populations by urbanicity (Table 3), in the urban areas, women who used newspaper/magazine [OR 1.12, 95%CI 1.02-1.24], used the radio [OR 1.37, 95%CI 1.13-1.65] or used television almost every day [OR 1.39, 95%CI 1.24-1.55], or went to the cinema at least once a month [OR 1.23, 95%CI 1.09-1.38] were more likely to attend at least eight ANC visits as compared to those who did not. In the rural areas, women who used newspaper/magazine [OR 1.43, 95%CI 1.31-1.57], used radio [OR 1.22, 95%CI 1.09-1.37], used television almost every day [OR 2.07, 95%CI 1.94-2.2], or went to the cinema at least once a month [OR 1.33, 95%CI 1.2-1.47], were also more likely to attend at least eight ANC visits as compared to those who did not (Figure 1).

For peer review only - http://bmjopen.bmj.com/site/about/guidelines.xhtml

Table 1: Socioeconomic, demographic, and media use characteristics by at least eight antenatal care visits among women who gave birth in the last five years, in the NFHS 2015-2016.

	At least eight antenatal care visits (N=29812) (%[1])
Total, N=187894 (100.0%)	20.3%
Newspaper/Magazine use	
Not every day (90.0%)	17.9%
Almost every day (9.9%)	40.8%
Radio use	
Not every day (96.6%)	19.8%
Almost every day (3.3%)	33.2%
Television use	
Not every day (44.9%)	9.5%
Almost every day (55%)	29.1%
Movie use	
Less than once a month (92.6%)	18.9%
At least once a month 7.4%)	37.1%
Age (years)	
15 to 19 (3.4%)	18.8%
20 to 24 (31.3%)	19.8%
25 to 29 (37.6%)	21.8%
30 to 34 (18.3%)	20.9%
35 to 39 (6.9%)	16.7%
40 to 44 (1.9%)	11.4%
45 to 49 (0.6%)	6.2%
Educational attainment	
No education (27.6%)	7.0%
Incomplete primary (5.9%)	14.4%
Complete primary (7.4%)	12.4%
Incomplete secondary (37.8%)	23.8%
Complete secondary 9.2%)	31.1%
Higher (12.0%)	38.9%
Current marital status	
Never married (0.1%)	16.8%
Married (98.6%)	20.3%
Widowed (0.7%)	17.5%
Divorced 0.1%)	24.1%
Separated (0.4%)	20.3%
Wealth	
1st quintile (Poorest) (23.4%)	5.7%
2nd quintile (21.2%)	13.4%
3rd quintile (19.9%)	21.7%
4th quintile (19.0%)	29.3%
5th quintile (Richest) (16.6%)	37.4%
Caste	
Schedule caste (21.4%)	18.2%
Schedule tribe (10.3%)	13.5%
Other backward class (44.0%)	19.5%
None of them/General (24.3%)	26.4%
Urbanicity	
Urban (29.6%)	31.4%
Rural (70.4%)	15.6%
Religion	
Hindu (79.0%)	19.8%
Muslim (15.9%)	20.4%
Christian (2.1%)	29.8%
Sikh (1.3%)	22.3%
Other (1.6%)	28.6%

[1]Indicates the weighted percentage of women with that particular socioeconomic, demographic, or media use characteristic who attended at least eight antenatal care visits.

For peer review only - http://bmjopen.bmj.com/site/about/guidelines.xhtml

Table 2: Media use characteristics by rural/urban strata among women who gave birth in the last five years in India

	Urban (N=46941)	*Rural (N=140953)*
	%[2]	%[2]
Total	100.0%	100.0%
Newspaper/Magazine use		
Not every day	78.7%	94.8%
Almost every day	21.3%	5.3%
Radio use		
Not every day	95.1%	97.3%
Almost every day	4.9%	2.8%
Television use		
Not every day	21.0%	54.9%
Almost every day	79.0%	45.1%
Movie use		
Less than once a month	85.5%	95.5%
At least once a month	14.5%	4.5%

[2]Indicates the weighted percentage of women in that particular strata using the specific type of media with that frequency.

Table 3: Crude Odds ratio (OR), adjusted OR and adjusted OR stratified by urbanicity, and 95% confidence intervals for the association between media use and making at least eight antenatal care visits among women who gave birth in the last five years, in the NFHS 2015-2016.

	At least eight antenatal care visits			
	**Crude**	**Adjusted***	**Adjusted* Urban**	**Adjusted* Rural**
	**OR (95% CI)**	**OR (95% CI)**	**OR (95% CI)**	**OR (95% CI)**
Newspaper/Magazine use				
Not every day (Reference)	1.00	1.00	1.00	1.00
Almost every day	3.14 (2.94-3.35)	1.26 (1.18-1.35)	1.12 (1.02-1.24)	1.43 (1.31-1.57)
Radio use				
Not every day (Reference)	1.00	1.00	1.00	1.00
Almost every day	2.02 (1.81-2.25)	1.29 (1.15-1.44)	1.37 (1.13-1.65)	1.22 (1.09-1.37)
Television use				
Not every day (Reference)	1.00	1.00	1.00	1.00
Almost every day	3.92 (3.72-4.14)	1.84 (1.74-1.95)	1.39 (1.24-1.55)	2.07 (1.94-2.2)
Movie use				
Less than once a month (Reference)	1.00	1.00	1.00	1.00
At least once a month	2.53 (2.34-2.74)	1.29 (1.19-1.4)	1.23 (1.09-1.38)	1.33 (1.2-1.47)

*Adjusted ORs are mutually adjusted for other media use variables as well as age, educational attainment, current marital status, wealth, caste, urbanicity and religion. OR= Odds ratio, CI= Confidence interval

For peer review only - http://bmjopen.bmj.com/site/about/guidelines.xhtml

Discussion

Media are one of the most valuable communication tools in public health. They have been, time and again, used to create awareness during public health emergencies. Mass media are a powerful tool when the target population is large and spread out, and in-person communication channels are unable to penetrate, especially through underserved rural areas.[21] Various forms of mass media have effectively educated and persuaded audiences to adopt healthy behaviors. Mass media have also been crucial in creating awareness about health-related programs and campaigns to promote healthy living and improve quality of life.[21]

Through this study, we were able to determine the associations between media use and ANC visits among Indian women during pregnancy. The descriptive characteristics established the popularity of different forms of mass media amongst Indian women. Overall, television appeared to be the most popular media source compared to newspapers/magazines, radio and watching movies in the cinema halls. According to Broadcast Audience Research Council India's recent survey, television is the preferred media of choice in India because of its affordability and its accessibility to free-to-air channels.[22] Furthermore, stratification by urbanicity suggests that television use is more prevalent in urban areas, compared to rural areas. This could be because urban areas in India have better access to facilities like electricity and cable. Other contributing factors could be higher literacy rates in urban areas due to better accessibility of schools and universities.[22,23]

In both rural and urban populations, almost every-day exposure to all types of mass media was positively associated with making at least eight ANC visits (Figure 1). These findings are in accord with studies from other developing countries such as Nepal, Bangladesh, and Uganda, where they also found positive associations between mass media exposure and ANC visits.[24,25] In Malawi, community-driven mass media campaigns in the rural areas resulted in increased uptake of maternal health services including ANC, which suggests that mass media can be an effective mode of intervention in limited-resource settings.[26] Interestingly, we observed that although the frequency of almost every day

For peer review only

use of television and other media was higher in the urban areas, the associations were stronger in the rural areas for all types of media, except for radio.

Our study shows that almost every-day use of newspaper/magazine is not very prevalent amongst Indian women, especially in rural areas. However, it is positively associated with making at least eight ANC visits, in both rural as well as urban regions, after adjusting for several SES indicators. Similarly, a large percentage of both rural and urban women do not use the radio almost every day or go to the cinema to watch movies every month. However, the associations of both types of media use with making at least eight ANC visits was positive in both rural and urban areas. These findings imply that using newspapers/magazines, radio, or movies at cinema halls as communication tools to create awareness about utilizing adequate ANC, might have a positive impact, however, only on a smaller fraction of women. Alternatively, our data indicate that television use is the most popular media source amongst women, in rural as well as urban areas. The positive association between television use and making at least eight ANC visits suggests that television could be an excellent media source to generate ANC awareness in women, especially in rural areas (Figure 2). Previous studies have reported that when television is used as a medium to create knowledge, knowledge gaps resulting from the communication flow of messages are less likely to occur.[27] Our study suggests that using television as a medium to create ANC awareness can have a similar effect on reducing differences in ANC behavior.

Evidently, television is the most effective media source to create awareness in our large target population. Nonetheless, even though only a small percentage of women, in both rural and urban areas, use other media sources, it is recommended to use a multimedia approach because, in big countries like India, even a small population percentage is significant.

Strengths and Limitations

A major strength of this study, based on nationally representative survey data, is its generalizability. Furthermore, these data can determine trends stratified by urbanicity,

For peer review only - http://bmjopen.bmj.com/site/about/guidelines.xhtml

states, SES, caste, and religion, which can further help to narrow down the at-risk populations and design tailored interventions. Insights from this study can inform health awareness programs in other developing nations with strong mass media traditions.

One of the main limitations of this study is using self-reported NFHS-4 data, making it prone to recall bias and under- or over-reporting of behaviors. Additionally, face-to-face administration of the survey by an interviewer increases the potential for interviewer bias. Studies have shown that personal interviews have poorer responses to sensitive questions with socially unacceptable responses.[28] The cross-sectional design of the data limits the ability to derive any causal relationships between the variables. Nonetheless, the strong association between media use and ANC visits suggest the potential promise of media in maternal health promotion.

It is also important to note that, in this study, we only determined the association between the four most common mass media sources and making at least eight ANC visits. However, other modes are also used to create ANC-related awareness in India, such as social media, billboards, cellphones, community theater, medical camps, door-to-door information by community healthcare workers, and pamphlet distributions.[18,29] NFHS-4 has no information about the type of ANC-related media content available to women in India. Thus, one can only speculate based on literature. Future studies should examine ANC-related media content to obtain a complete picture of the kind of information encouraging such behaviors. Moreover, other sources of media such as the internet, computers, and smartphones are gaining popularity in India. Future surveys should also include information regarding their use and their implications on health-related behaviors.

Conclusion

In India, there is a need for increased awareness about obtaining adequate ANC visits. Positive associations between different forms of mass media with making at least eight ANC visits provide evidence that exposure to media may encourage positive pregnancy behaviors. In both rural and urban India, the popularity of television and its association with making at least eight ANC visits is stronger than other forms of media. ANC related

For peer review only

health awareness campaigns in the country can use these findings to allocate resources to the appropriate forms of mass media to encourage healthy behaviors. Moreover, clearly, using mass media, especially television, can promote maternal health at a scale, unlike other more labor-intensive approaches.

Author Contributions DD and KV conceived and designed the study. RP and MAB contributed to the design of the study. DD analyzed and interpreted the data and wrote the first draft of the manuscript. DD, RP, MAB and KV contributed to the interpretation of the data and revised the manuscript for important intellectual content. All authors provided critical feedback on the manuscript and agree to be accountable for all aspects of the work.

Funding This research was supported by funding from the Bill & Melinda Gates Foundation (Viswanath, PI), grant number INV-005090. The funders had no role in the study design; in the collection, analysis and interpretation of the data; in the writing of the report; and in the decision to submit the paper for publication.

Competing Interests None declared

Patient and public involvement Patients and/or the public were not involved in the design, or conduct, or reporting, or dissemination plans of this research.

Patient consent for publication Not required.

Ethics approval the NFHS-4 was directed by the Ministry of Family Health and Welfare of the Government of India and administered by the International Institute for Population Sciences in Mumbai, India. Approval for the use of these data for this study was granted by the Institutional Review Board of Tufts University School of Medicine/Tufts Medical Center.

Provenance and peer review Not commissioned; externally peer reviewed.

Data availability statement Data are available in a public, from the Demographic and Health Surveys Program.

For peer review only - http://bmjopen.bmj.com/site/about/guidelines.xhtml

Bibliography

1 World Health Organization. *WHO Recommendation on Antenatal care for positive pregnancy experience*. 2016. doi:ISBN 978 92 4 154991 2

2 Kumar G, Choudhary TS, Srivastava A, *et al.* Utilisation, equity and determinants of full antenatal care in India: analysis from the National Family Health Survey 4. *BMC Pregnancy Childbirth* 2019;**19**:327. doi:10.1186/s12884-019-2473-6

3 Dixit P, Dwivedi LK, Ram F. Strategies to Improve Child Immunization via Antenatal Care Visits in India: A Propensity Score Matching Analysis. *PLoS One* 2013;**8**:e66175. doi:10.1371/journal.pone.0066175

4 World Health Organization. New guidelines on antenatal care for a positive pregnancy experience. World Health Organization 2017. https://www.who.int/reproductivehealth/news/antenatal-care/en/ (accessed 1 Apr 2020).

5 Doku DT, Neupane S. Survival analysis of the association between antenatal care attendance and neonatal mortality in 57 low- And middle-income countries. *Int J Epidemiol* 2017;**46**:1668–77. doi:10.1093/ije/dyx125

6 UNICEF. Antenatal Care - UNICEF data. 2019. https://data.unicef.org/topic/maternal-health/antenatal-care/# (accessed 5 May 2020).

7 Viswanath K, Ramanadhan S, Kontos EZ. Mass Media. In: *Macrosocial Determinants of Population Health*. New York, NY: : Springer New York 2007. 275–94. doi:10.1007/978-0-387-70812-6_13

8 Hornik R, Yanovitzky I. Using theory to design evaluations of communication campaigns: The case of the national youth anti-drug media campaign. *Commun Theory* 2003;**13**:204–24. doi:10.1111/j.1468-2885.2003.tb00289.x

9 Wakefield MA, Loken B, Hornik RC. Use of mass media campaigns to change health behaviour. *Lancet* 2010;**376**:1261–71. doi:10.1016/S0140-6736(10)60809-4

10 Fishbein M, Ajzen I. Predicting and changing behavior: The reasoned action approach. *Predict Chang Behav Reason Action Approach* 2011;:1–518. doi:10.4324/9780203838020

11 Jones G, Steketee RW, Black RE, *et al.* How many child deaths can we prevent this year? *Lancet* 2003;**362**:65–71. doi:10.1016/S0140-6736(03)13811-1

12 Kuhnt J, Vollmer S. Antenatal care services and its implications for vital and health outcomes of children: Evidence from 193 surveys in 69 low-income and middle-income countries. *BMJ Open* 2017;**7**. doi:10.1136/bmjopen-2017-017122

13 Ghosh R. Child mortality in India: A complex situation. *World J Pediatr* 2012;**8**:11–8. doi:10.1007/s12519-012-0331-y

14 World Health Organization G, Fund UNC, Fund UNP, *et al. Trends in maternal mortality 2000 to 2017: estimates by WHO, UNICEF, UNFPA, World Bank Group and the United Nations Population Division.* 2019.

For peer review only - http://bmjopen.bmj.com/site/about/guidelines.xhtml

15 UNICEF. *Children , food and nutrition: growing well in a changing world*. 2019.

16 Murukutla N, Yan H, Wang S, *et al.* Cost-effectiveness of a smokeless tobacco control mass media campaign in India. *Tob Control* 2018;**27**:547–51. doi:10.1136/tobaccocontrol-2016-053564

17 Curtis V. Explaining the outcomes of the 'Clean India' campaign: Institutional behaviour and sanitation transformation in India. *BMJ Glob Heal* 2019;**4**:e001892. doi:10.1136/bmjgh-2019-001892

18 JAN ANDOLAN-Guidelines. https://icds-wcd.nic.in/nnm/NNM-Web-Contents/LEFT-MENU/Guidelines/JanAndolanGuidelines-English.pdf (accessed 5 May 2020).

19 National Family Health Survey. National Family Health Survey , India. 2017. http://rchiips.org/nfhs/NFHS-4Reports/India.pdf (accessed 10 May 2020).

20 Saroha E, Altarac M, Sibley LM. Caste and Maternal Health Care Service Use Among Rural Hindu Women in Maitha, Uttar Pradesh, India. *J Midwifery Women's Heal* 2008;**53**:41–7. doi:10.1016/j.jmwh.2008.05.002

21 unite for sight. Health Communication Course: Module 5 - The Role of Media in Health Promotion. Unite for Sight. 2010.https://www.uniteforsight.org/health-communication-course/module5 (accessed 3 Dec 2019).

22 BARC India. The Changing Faces of TV in India. https://www.barcindia.co.in/resources/The Changing Face of TV in India.pdf (accessed 7 May 2020).

23 Implementation P, Office NS. Key Indicators of Household Social Consumption on Education in India Government of India Ministry of Statistics and Programme Implementationin GoIStats GoIStats goistats. 2017. http://www.mospi.gov.in/sites/default/files/publication_reports/KI_Education_75th_Final.pdf (accessed 8 Dec 2019).

24 Acharya D, Khanal V, Singh JK, *et al.* Impact of mass media on the utilization of antenatal care services among women of rural community in Nepal. *BMC Res Notes* 2015;**8**:345. doi:10.1186/s13104-015-1312-8

25 Bbaale E. Factors influencing the utilisation of antenatal care content in Uganda. *Australas Med J* 2011;**4**:516–26. doi:10.4066/AMJ.2011.849

26 Zamawe COF, Banda M, Dube AN. The impact of a community driven mass media campaign on the utilisation of maternal health care services in rural Malawi. *BMC Pregnancy Childbirth* 2016;**16**:21. doi:10.1186/s12884-016-0816-0

27 Viswanath K, Finnegan JR. The Knowledge Gap Hypothesis: Twenty-Five Years Later. In: *Annals of the International Communication Association*. 1996. 187–228. doi:10.1080/23808985.1996.11678931

28 Salazar MK. Interviewer bias: How it affects survey research. *AAOHN J* 1990;**38**:567–72. doi:10.1177/216507999003801203

29 Chandra A, Sarkar S, Adinarayanan S, *et al.* Impact of health awareness campaign in improving the perception of the community about palliative care: A

For peer review only - http://bmjopen.bmj.com/site/about/guidelines.xhtml

pre- and post-intervention study in rural Tamil Nadu. *Indian J Palliat Care* 2016;**22**:467–76. doi:10.4103/0973-1075.191832

Figure Legends

Figure 1: Association between different types of media use and making at least eight antenatal care visits, stratified by urbanicity.

Figure 2: Association between television use and making at least eight antenatal care visits, stratified by urbanicity. (Error bars = 95% confidence intervals)

For peer review only

For peer review only - http://bmjopen.bmj.com/site/about/guidelines.xhtml

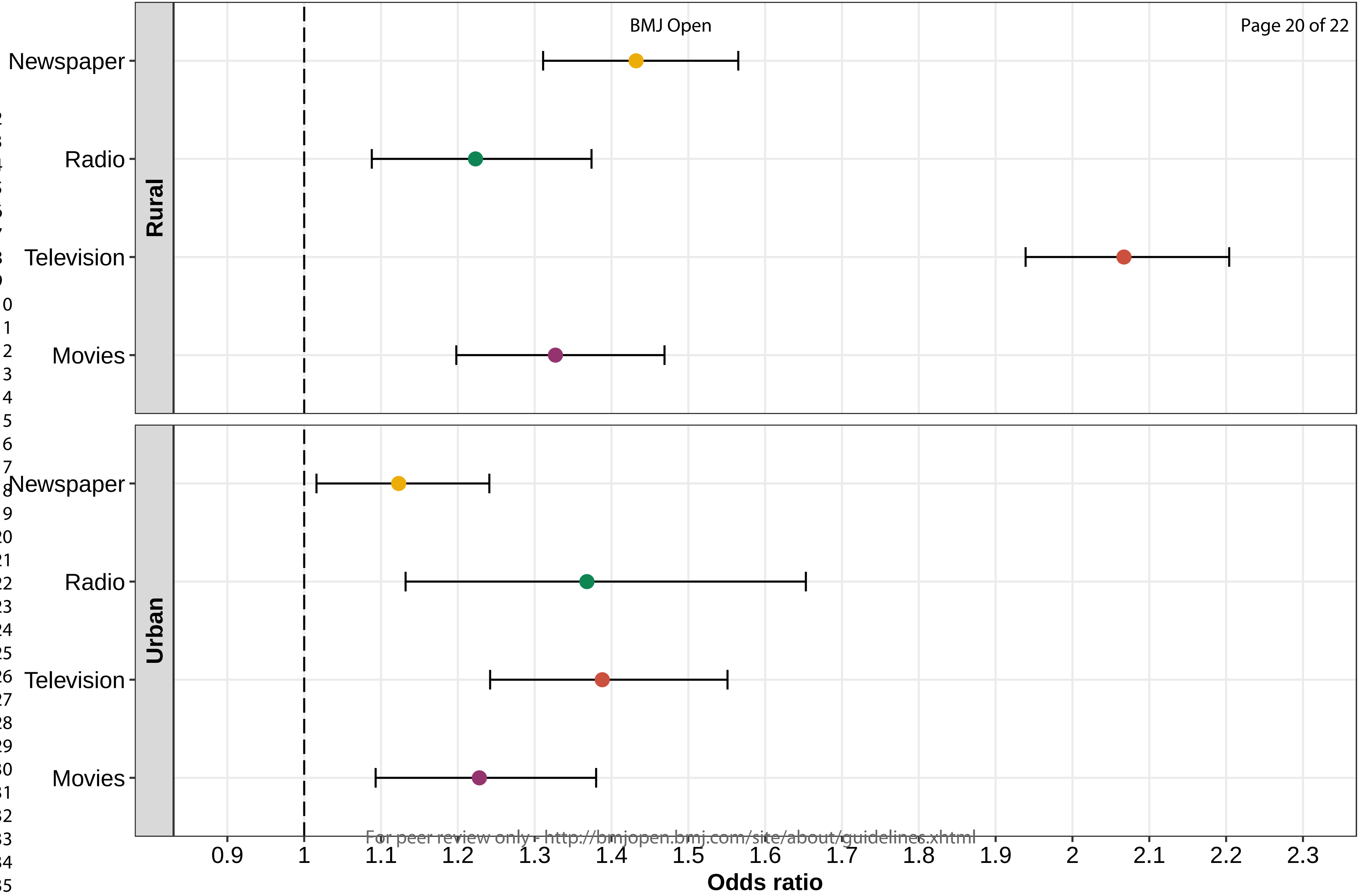
Rural
Urban
Newspaper
Radio
Television
Movies
0.9
1
1.1
1.2
1.3
1.4
1.5
1.6
1.7
1.8
1.9
2
2.1
2.2
2.3
Odds ratio

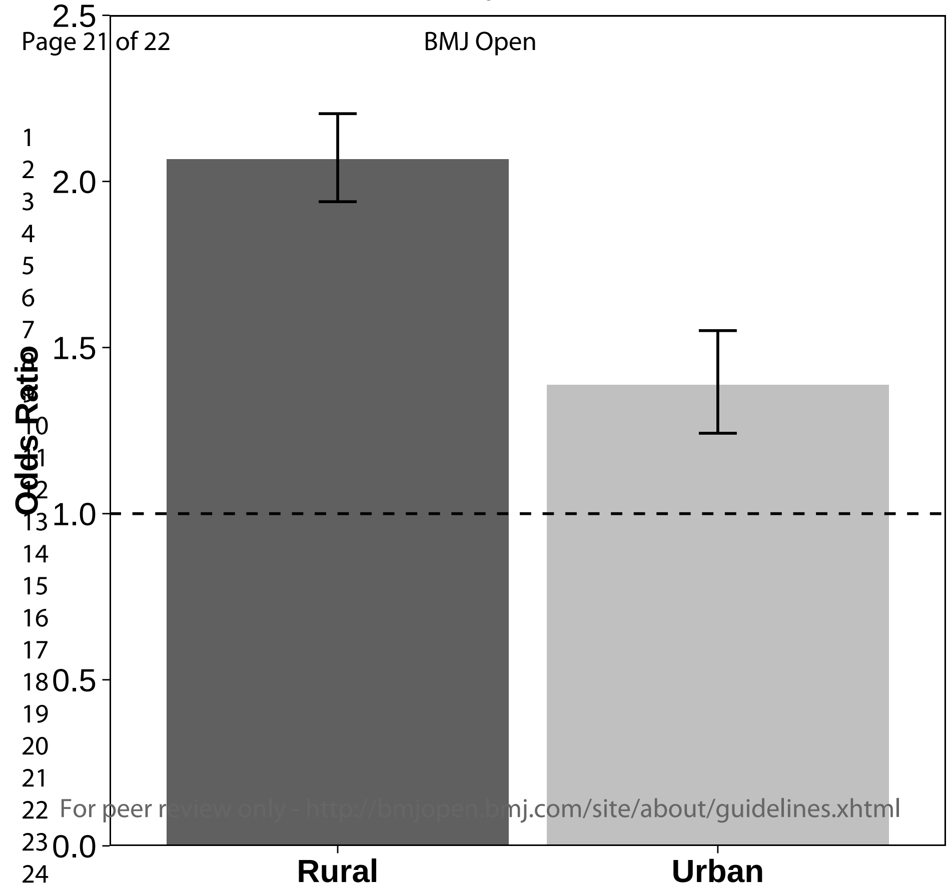

For peer review only - http://bmjopen.bmj.com/site/about/guidelines.xhtml

STROBE 2007 (v4) Statement—Checklist of items that should be included in reports of *cross-sectional studies*

**Section/Topic**	**Item #**	**Recommendation**	**Reported on page #**
**Title and abstract**	1	(*a*) Indicate the study's design with a commonly used term in the title or the abstract	2
		(*b*) Provide in the abstract an informative and balanced summary of what was done and what was found	2
**Introduction**			
Background/rationale	2	Explain the scientific background and rationale for the investigation being reported	5
Objectives	3	State specific objectives, including any prespecified hypotheses	6
**Methods**			
Study design	4	Present key elements of study design early in the paper	6
Setting	5	Describe the setting, locations, and relevant dates, including periods of recruitment, exposure, follow-up, and data collection	6
Participants	6	(*a*) Give the eligibility criteria, and the sources and methods of selection of participants	6
Variables	7	Clearly define all outcomes, exposures, predictors, potential confounders, and effect modifiers. Give diagnostic criteria, if applicable	7
Data sources/ measurement	8*	For each variable of interest, give sources of data and details of methods of assessment (measurement). Describe comparability of assessment methods if there is more than one group	6
Bias	9	Describe any efforts to address potential sources of bias	*6*
Study size	10	Explain how the study size was arrived at	6
Quantitative variables	11	Explain how quantitative variables were handled in the analyses. If applicable, describe which groupings were chosen and why	7
Statistical methods	12	(*a*) Describe all statistical methods, including those used to control for confounding	8
		(*b*) Describe any methods used to examine subgroups and interactions	8
		(*c*) Explain how missing data were addressed	6
		(*d*) If applicable, describe analytical methods taking account of sampling strategy	n/a
		(*e*) Describe any sensitivity analyses	n/a
**Results**			

For peer review only

Participants	13*	(a) Report numbers of individuals at each stage of study—eg numbers potentially eligible, examined for eligibility, confirmed eligible, included in the study, completing follow-up, and analysed	6
		(b) Give reasons for non-participation at each stage	6
		(c) Consider use of a flow diagram	n/a
Descriptive data	14*	(a) Give characteristics of study participants (eg demographic, clinical, social) and information on exposures and potential confounders	9
		(b) Indicate number of participants with missing data for each variable of interest	6
Outcome data	15*	Report numbers of outcome events or summary measures	9
Main results	16	(*a*) Give unadjusted estimates and, if applicable, confounder-adjusted estimates and their precision (eg, 95% confidence interval). Make clear which confounders were adjusted for and why they were included	9
		(*b*) Report category boundaries when continuous variables were categorized	n/a
		(*c*) If relevant, consider translating estimates of relative risk into absolute risk for a meaningful time period	n/a
Other analyses	17	Report other analyses done—eg analyses of subgroups and interactions, and sensitivity analyses	9
**Discussion**			
Key results	18	Summarise key results with reference to study objectives	12
Limitations	19	Discuss limitations of the study, taking into account sources of potential bias or imprecision. Discuss both direction and magnitude of any potential bias	14
Interpretation	20	Give a cautious overall interpretation of results considering objectives, limitations, multiplicity of analyses, results from similar studies, and other relevant evidence	13
Generalisability	21	Discuss the generalisability (external validity) of the study results	13
**Other information**			
Funding	22	Give the source of funding and the role of the funders for the present study and, if applicable, for the original study on which the present article is based	15

*Give information separately for cases and controls in case-control studies and, if applicable, for exposed and unexposed groups in cohort and cross-sectional studies.

Note: An Explanation and Elaboration article discusses each checklist item and gives methodological background and published examples of transparent reporting. The STROBE checklist is best used in conjunction with this article (freely available on the Web sites of PLoS Medicine at http://www.plosmedicine.org/, Annals of Internal Medicine at http://www.annals.org/, and Epidemiology at http://www.epidem.com/). Information on the STROBE Initiative is available at www.strobe-statement.org.

For peer review only

BMJ Open

The Association Between Different Types of Mass Media and Antenatal Care Visits in India: A Cross-Sectional Study from The National Family Health Survey (2015-2016)

Journal:	*BMJ Open*
Manuscript ID	bmjopen-2020-042839.R1
Article Type:	Original research
Date Submitted by the Author:	17-Nov-2020
Complete List of Authors:	Dhawan, Dhriti; Dana-Farber Cancer Institute, Medical Oncology<br>Pinnamaneni, Ramya; Harvard University T H Chan School of Public Health,<br>Bekalu, Mesfin; Harvard University T H Chan School of Public Health,<br>Viswanath, Kasisomayajula; Harvard University T H Chan School of Public Health, ; Dana-Farber Cancer Institute, Medical Oncology
**Primary Subject Heading**:	Public health
Secondary Subject Heading:	Communication
Keywords:	EPIDEMIOLOGY, OBSTETRICS, PUBLIC HEALTH

SCHOLARONE™
Manuscripts

I, the Submitting Author has the right to grant and does grant on behalf of all authors of the Work (as defined in the below author licence), an exclusive licence and/or a non-exclusive licence for contributions from authors who are: i) UK Crown employees; ii) where BMJ has agreed a CC-BY licence shall apply, and/or iii) in accordance with the terms applicable for US Federal Government officers or employees acting as part of their official duties; on a worldwide, perpetual, irrevocable, royalty-free basis to BMJ Publishing Group Ltd ("BMJ") its licensees and where the relevant Journal is co-owned by BMJ to the co-owners of the Journal, to publish the Work in this journal and any other BMJ products and to exploit all rights, as set out in our licence.

The Submitting Author accepts and understands that any supply made under these terms is made by BMJ to the Submitting Author unless you are acting as an employee on behalf of your employer or a postgraduate student of an affiliated institution which is paying any applicable article publishing charge ("APC") for Open Access articles. Where the Submitting Author wishes to make the Work available on an Open Access basis (and intends to pay the relevant APC), the terms of reuse of such Open Access shall be governed by a Creative Commons licence – details of these licences and which Creative Commons licence will apply to this Work are set out in our licence referred to above.

Other than as permitted in any relevant BMJ Author's Self Archiving Policies, I confirm this Work has not been accepted for publication elsewhere, is not being considered for publication elsewhere and does not duplicate material already published. I confirm all authors consent to publication of this Work and authorise the granting of this licence.

For peer review only

The Association Between Different Types of Mass Media and Antenatal Care Visits in India: A Cross-Sectional Study from The National Family Health Survey (2015-2016)

Correspondence to:

Dhriti Dhawan, PhD, MPH;

Dana-Farber Cancer Institute

450 Brookline Avenue, LW 601

Boston, MA 02215-5450
Tel: (617) 632-6586
Fax: (617) 582-8728

dhriti_dhawan@dfci.harvard.edu

Co-Authors:

1. **Dhriti Dhawan, PhD, MPH;**

   Department: Medical Oncology

   Institution: Dana-Farber Cancer Institute, Boston, MA, USA,

   Full address: 450 Brookline Ave. Boston, MA 02215

2. **Ramya Pinnamaneni, MBBS, MPH;**

   Department: Social and Behavioral Sciences

   Institution: Harvard T.H Chan School of Public Health, Boston, MA, USA,

   Full address: 450 Brookline Ave. Boston, MA 02215

3. **Mesfin Bekalu, PhD;**

   Department: Social and Behavioral Sciences

   Institution: Harvard T.H Chan School of Public Health, Boston, MA, USA,

   Full address: 450 Brookline Ave. Boston, MA 02215

4. **Kasisomayajula Viswanath, PhD**

   Department: Social and Behavioral Sciences

   Institution: Harvard T.H Chan School of Public Health, Boston, MA, USA,

   Full address: 450 Brookline Ave. Boston, MA 02215

For peer review only

Abstract

Objective To generate evidence for the association between different types of mass media and Ante Natal care (ANC) visits in India.

Design A cross-sectional study design, analyzing data from India's National Family Health Survey 4 (2015-16) (NFHS-4).

Setting Rural and Urban India.

Participants From NFHS-4, women who had given birth in the last five years before survey administration were included in this study. Women with missing information about their number of ANC visits and their caste were excluded, leaving 187,894 women in the final analytical sample.

Primary outcome measures Logistic regression analysis was conducted to determine the association between ANC utilization with mass media exposure.

Results Overall, our study showed that high exposure to all four types of mass media was positively associated with making at least eight ANC visits. In rural India, women who had high exposure to newspaper/magazine [adjusted OR (aOR) 1.43, 95% CI 1.31-1.57], radio [aOR 1.22, 95% CI 1.09- 1.37], television [aOR 2.07, 95% CI 1.94-2.2], and movies [aOR 1.33, 95% CI 1.2-1.47] were more likely to make at least eight ANC visits. In Urban India, women who had high exposure to newspaper/magazine [aOR 1.12, 95% CI 1.02-1.24], radio [aOR 1.37, 95% CI 1.13-1.65], television [aOR 1.39, 95% CI 1.24-1.55], and movies [aOR 1.23, 95% CI 1.09-1.38] were more likely to make at least eight ANC visits.

Conclusions Our findings emphasize the need for increased awareness about obtaining adequate ANC visits in India, to improve maternal, neonatal, and child health outcomes. Our study highlights that television penetration is broader than other forms of media and has the potential to create awareness about health in both urban and rural populations. These findings can inform ANC related health awareness campaigns in the country to allocate resources to the appropriate media sources to encourage healthy behaviors.

Keywords Antenatal care, Maternal health, Child health, Health communication, LMIC

Word count 3280

For peer review only

Article Summary

Strengths and limitations of this study

- Our study provides a novel insight on the association between mass media exposure and making at least eight ANC visits in India, with central focus on mass media exposure.
- Our results are derived from a nationally representative survey and are generalizable to women in India.
- The data enable us to determine trends stratified by urbanicity, states, Socioeconomic Status (SES), caste, and religion, which can further help to narrow down the at-risk populations and design customized interventions.
- The cross-sectional design of the data limits the ability to derive any causal inferences on the relationship between the variables. Nonetheless, the strong associations between media use and ANC visits suggest the potential promise of media in maternal health promotion.
- The dataset has no information about the type of ANC-related media content available to women in India.

For peer review only

Introduction

Ante Natal Care (ANC) is widely acknowledged as a critical tool in reducing stillbirths, complications in pregnancy, and promoting positive pregnancy experience.[1] Components of ANC are risk identification, prevention and management of pregnancy-related or concurrent diseases, and maternal health education and health promotion.[1] ANC is conducive for a healthy pregnancy and improves the chances of survival for the mother as well as for the child. It has also been shown to support the growth and development of the child until the age of five years.[2] Women who utilize ANC services are more likely to use other essential services such as counseling for breastfeeding, nutrition, postpartum family planning as well as childhood immunization.[3]

Before 2016, the World Health Organization (WHO) recommended at least four ANC visits during normal pregnancy and that the first visit be completed within the first 8-12 weeks of conception.[1] However, in 2016, the WHO changed its recommendation to a minimum of eight ANC visits.[1] This was because, compared to four visits, eight or more ANC visits have the potential to reduce perinatal deaths by up to 8 per 1000 births, as it provides more opportunities for detecting and treating potential complications.[4] Despite these recommendations, women of low- and middle-income countries have low ANC coverage.[1,5] According to UNICEF, there are wide disparities in ANC coverage across countries, with the lowest levels in sub-Saharan Africa and South Asia.[6] There is an urgent need to create awareness about the benefits of utilizing ANC services and support interventions aimed towards increasing their utilization to improve maternal, neonatal as well as child health. We need strategies that can promote ANC service utilization at scale.

Mass media campaigns

Mass media are critical tools in promoting health through two key strategies: one, their widespread penetration promotes broad reach to key audiences across boundaries, and two, exposure to specific messages in the media are known to shape public knowledge, attitudes, beliefs and behaviors.[7] Routine use of media can influence both pro- and anti-healthy behaviors.[8] Media campaigns have been extensively used over the past few

For peer review only - http://bmjopen.bmj.com/site/about/guidelines.xhtml

decades to induce behavioral changes in populations, especially in the context of substance use, such as tobacco, alcohol, and illicit drugs.

Creating awareness through mass media can encourage positive or discourage negative health-related behaviors across populations, through direct and indirect pathways.[8] These behavioral changes occur passively as a result of routine media use.[9] Mass media campaigns can invoke cognitive or emotional responses at an individual level by informing the population about the benefits and risks of a particular health-related behavior. These campaigns frequently associate emotions with achieving change, strengthening the probability of alteration, and increasing the likelihood of adopting new behaviors.[10] For example, as a part of India's Pradhan Mantri Surakshit Matritva Abhiyan (PMSMA) campaign, public service announcements (PSA) were released. These PSAs have prompted pregnant women as well as their family members to seek ANC during pregnancy, informed them about the benefits of these visits, and highlighted that these visits will be free.[11] The WHO commended India for its groundbreaking progress in reducing maternal mortality and credited the PMSMA campaign for its contribution towards this achievement.[12]

Many low-income countries face poor child survival before five years of age because of inadequate treatment of diarrhea, non-vaccination for preventable diseases, inadequate ANC, and inadequate breastfeeding.[13,14] Mass media campaigns have been used to spread awareness and educate populations about each of these causes, and have resulted in mixed evidence for success.[9] Television and radio are the most popular media for creating awareness amongst large audiences. However, print media, such as magazines and newspapers, and outdoor media, such as billboards and posters, have also proven effective.[9] In India, 65.2% of households own a television and 8.1% of households own a radio. Moreover, 66% of women in rural India and 92% women in urban India have regular exposure to some kind of mass media.[15] Broadcasting important messages related to ANC through mass media may help in increasing awareness at the population level.[2]

For peer review only - http://bmjopen.bmj.com/site/about/guidelines.xhtml

Antenatal Care in India

Our study examines the association between mass media exposure and ANC visits in India. Indian women carry a heavy burden of poor nutritional status and infections, which is associated with high child mortality as well as maternal mortality.[16] In 2017, India recorded 35,000 maternal deaths- second highest in the world.[17] In 2019, India recorded 882,000 children under-five deaths and 549,000 neonatal deaths- highest in the world.[18] ANC visits are of high importance because they can help prevent adverse effects on the health of women and children, and reduce the risk of under-five and neonatal deaths.[14] To encourage women to access ANC in India, public healthcare facilities provide these services for free.[15] Moreover, women of low socioeconomic status are given cash incentives for giving birth in a government or an accredited private healthcare facility through schemes such as the *Janani Suraksha Yojana* (JSY). This scheme also incentivizes healthcare workers to facilitate ANC visits along with institutional births.[19] Studies have shown that JSY has significantly increased ANC visits in India.[20] Despite such incentives, only 51.6% pregnant Indian women received four or more ANC visits, which is lower than the global average of 62% during 2010-2016.[2,6]

Many intervention programs in India use mass media as one of the communication tools to create health-related awareness.[21,22] For example, the National Nutrition Mission of India (*POSHAN Abhiyaan*) plans to utilize various platforms including mass media to inspire a people's movement, *Jan Andolan*, by creating awareness about nutrition and ensuring the engagement of the population at different levels.[23] The objective of our study is to generate evidence for the association between different types of mass media and ANC visits in India. Specifically, within ANC, we tested the association of the self-reported number of ANC visits with the level of exposure to four types of mass media: newspapers/magazines, radio, television, and movies.

For peer review only - http://bmjopen.bmj.com/site/about/guidelines.xhtml

Methods

Data source

This analysis utilizes individual-level data from India's National Family Health Survey 4 (NFHS-4), 2015-16.[15] The NFHS series are nationally representative cross-sectional surveys that provide data on a range of demographic, socioeconomic, maternal and child health outcomes, reproductive health, and family planning. NFHS-4 gathered information from 601,509 households, 699,686 women, and 112,122 men, with a response rate of 97%. Of the women, 190,898 women had given birth in the last five years. 1,854 women were missing information about their number of ANC visits during pregnancy, and 1,150 women were missing information about their caste. These women were excluded from the study, thereby leaving 187,894 women in the analytical sample.

Sample design

The NFHS-4 used a two-stage stratified sampling design. The 2011 census served as the sampling frame for the selection of primary sampling units (PSUs). The samples were stratified by rural and urban areas, and villages were PSUs in the rural areas, and census enumeration blocks were PSUs in the urban areas. Before the main survey, for each PSU, a household mapping and listing operation was carried out. Selected PSUs with at least 300 households were further segmented into 100-150 household segments. Systematic sampling was used to randomly select two of the segments, with probability proportional to segment size. Hence, an NFHS-4 cluster consists of either a PSU or a segment of a PSU. Twenty-two households were randomly selected with systematic sampling in every selected rural and urban cluster.

Outcome measure

For their most recent pregnancy in the last five years, women were asked: "How many times did you receive antenatal care during this pregnancy?". This was a continuous variable, which was recoded to a binary variable as women who had eight or more antenatal care visits and women who did not.

For peer review only - http://bmjopen.bmj.com/site/about/guidelines.xhtml

Exposures

Women were asked “Do you read a newspaper or magazine almost every day, at least once a week, less than once a week or not at all?”, “Do you listen to the radio almost every day, at least once a week, less than once a week or not at all?”, and “Do you watch television almost every day, at least once a week, less than once a week or not at all?”. These were categorical variables with the four given option categories. These variables were recoded to dichotomous variables with response options “High exposure” (for women who responded “Almost every day”) and “Low Exposure” (combining women who responded “At least once a week”, “Less than once a week” or “Not at all”). They were also asked, “Do you usually go to a cinema hall or theatre to see a movie at least once a month?” with response options “Yes” and “No”.

Covariates

The age of the participants was categorized in increments of five years and ranged from 15 to 49 years of age. Educational attainment of the participants was categorized per the Indian educational system: No education, Incomplete primary education, Complete primary education, Incomplete secondary education, Complete secondary education, and Higher. Participants were grouped according to their marital status: Never married, Married, Widowed, Divorced, and Separated. In India, wealth has been established as a valid measure of socioeconomic status (SES). It can be defined as a measure of housing characteristics and possession of materials. Using principal component analysis, a wealth score was assigned to each participating household. Each person in the household was assigned a household score and ranked according to it. The distribution was divided into quintiles. Wealth of the participants was categorized accordingly.[15] The Caste system in India has contributed to the social marginalization of people who belong to certain groups and may potentially influence access to healthcare including maternal healthcare.[24] NFHS reports four categories: Scheduled Castes, Scheduled Tribes, Other Backward Classes, and General. The urbanicity of the participants was categorized as rural or urban. The religion of the participants was categorized as Hindu, Muslim, Christian, Sikh, and other.

For peer review only - http://bmjopen.bmj.com/site/about/guidelines.xhtml

Statistical analysis

Frequencies of key outcome and exposure variables as well as the covariates were obtained to describe the study sample. Participants with "Do not know" responses for our variables of interest were recoded to missing and excluded from the present study. Logistic regression analysis was conducted to determine the association between our outcome variable: "At least eight antenatal care visits", and our respective exposure variables: "Newspaper/Magazine", "Radio", "Television", or "Movie". Multivariable logistic regression was conducted with each of the media variables while adjusting for other media variables as well as the covariates. The data were analyzed and weighted using R version 3.6.1 (2019-07-05). Survey weights and clustering within PSUs were included in the analysis and the survey-weighted logistic regression was carried with the R survey package.

Patient and public involvement

Patients and/or the public were not involved in the design, or conduct, or reporting, or dissemination plans of this research.

Results

Based on our analysis of the NFHS-4 survey, Indian women reported making an average of 4.8 ANC visits (SE ±0.03) during pregnancy. As per WHO's revised recommendation in 2016, only 20.3% of pregnant Indian women received at least eight ANC visits. 9.9%, 3.3%, and 55.1% of our target population had high exposure to newspaper/magazine, radio, and television, respectively, and 7.4% of our target population went to the cinema to watch a movie at least once a month (Table 1). Needless to say, television penetration and reach are broad compared to other media. The percentage of women making at least eight ANC visits increased with an increase in the indicators of SES such as education attainment after complete primary education, and wealth. Comparing media exposure characteristics amongst rural and urban women, each type of media exposure was higher in an urban context. The sample size in each cell fulfilled the conditions to permit the use of multivariable logistic regressions (Table 2).

For peer review only - http://bmjopen.bmj.com/site/about/guidelines.xhtml

Media and at least eight ANC visits

After adjusting for age, educational attainment, current marital status, wealth, caste, religion, and other media variables, women who had high exposure to newspaper/magazine [aOR 1.26, 95%CI 1.18-1.35], radio [aOR 1.29, 95% CI 1.15-1.44], television [aOR 1.84, 95%CI 1.74-1.95], or went to the cinema to see a movie at least once a month [aOR 1.29, 95%CI 1.19-1.4] were more likely to receive at least eight ANC visits as compared to those who did not (Table 3).

Upon stratification of the populations by urbanicity (Table 3), in the urban areas, women who had high exposure to newspaper/magazine [aOR 1.12, 95%CI 1.02-1.24], radio [aOR 1.37, 95%CI 1.13-1.65] or television [aOR 1.39, 95%CI 1.24-1.55], or went to the cinema at least once a month [aOR 1.23, 95%CI 1.09-1.38] were more likely to attend at least eight ANC visits as compared to those who did not. In the rural areas, women who had high exposure to newspaper/magazine [aOR 1.43, 95%CI 1.31-1.57], radio [aOR 1.22, 95%CI 1.09-1.37], television [aOR 2.07, 95%CI 1.94-2.2], or went to the cinema at least once a month [aOR 1.33, 95%CI 1.2-1.47], were also more likely to attend at least eight ANC visits as compared to those who did not (Figure 1).

For peer review only

For peer review only - http://bmjopen.bmj.com/site/about/guidelines.xhtml

Table 1: Socioeconomic, demographic, and media exposure characteristics by at least eight antenatal care visits among women who gave birth in the last five years, in the NFHS 2015-2016.

	At least eight antenatal care visits (N=29812)
	(%[1])
**Total, N=187894 (100.0%)**	20.3%
**Newspaper/Magazine**	
Low Exposure (90.0%)	17.9%
High Exposure (9.9%)	40.8%
**Radio**	
Low Exposure (96.6%)	19.8%
High Exposure (3.3%)	33.2%
**Television**	
Low Exposure (44.9%)	9.5%
High Exposure (55%)	29.1%
**Movie**	
Less than once a month (92.6%)	18.9%
At least once a month 7.4%)	37.1%
**Age (years)**	
15 to 19 (3.4%)	18.8%
20 to 24 (31.3%)	19.8%
25 to 29 (37.6%)	21.8%
30 to 34 (18.3%)	20.9%
35 to 39 (6.9%)	16.7%
40 to 44 (1.9%)	11.4%
45 to 49 (0.6%)	6.2%
**Educational attainment**	
No education (27.6%)	7.0%
Incomplete primary (5.9%)	14.4%
Complete primary (7.4%)	12.4%
Incomplete secondary (37.8%)	23.8%
Complete secondary 9.2%)	31.1%
Higher (12.0%)	38.9%
**Current marital status**	
Never married (0.1%)	16.8%
Married (98.6%)	20.3%
Widowed (0.7%)	17.5%
Divorced 0.1%)	24.1%
Separated (0.4%)	20.3%
**Wealth**	
1st quintile (Poorest) (23.4%)	5.7%
2nd quintile (21.2%)	13.4%
3rd quintile (19.9%)	21.7%
4th quintile (19.0%)	29.3%
5th quintile (Richest) (16.6%)	37.4%
**Caste**	
Schedule caste (21.4%)	18.2%
Schedule tribe (10.3%)	13.5%
Other backward class (44.0%)	19.5%
None of them/General (24.3%)	26.4%
**Urbanicity**	
Urban (29.6%)	31.4%
Rural (70.4%)	15.6%
**Religion**	
Hindu (79.0%)	19.8%
Muslim (15.9%)	20.4%
Christian (2.1%)	29.8%
Sikh (1.3%)	22.3%
Other (1.6%)	28.6%

[1]Indicates the weighted percentage of women with that particular socioeconomic, demographic, or media exposure characteristic who attended at least eight antenatal care visits.

For peer review only - http://bmjopen.bmj.com/site/about/guidelines.xhtml

Table 2: Media exposure characteristics by rural/urban strata among women who gave birth in the last five years in India

	Urban (N=46941)	*Rural (N=140953)*
	%[2]	%[2]
Total	100.0%	100.0%
Newspaper/Magazine		
Low Exposure	78.7%	94.8%
High Exposure	21.3%	5.3%
Radio		
Low Exposure	95.1%	97.3%
High Exposure	4.9%	2.8%
Television		
Low Exposure	21.0%	54.9%
High Exposure	79.0%	45.1%
Movie		
Less than once a month	85.5%	95.5%
At least once a month	14.5%	4.5%

[2]Indicates the weighted percentage of women in that strata with exposure to the specific type of media with that frequency.

Table 3: Crude Odds ratio (OR), adjusted OR (aOR) and aOR stratified by urbanicity, and 95% confidence intervals (95% CI) for the association between media exposure and making at least eight antenatal care visits among women who gave birth in the last five years, in the NFHS 2015-2016.

	At least eight antenatal care visits			
			Urban	Rural
	Crude OR (95% CI)	aOR* (95% CI)	aOR* (95% CI)	aOR* (95% CI)
Newspaper/Magazine				
Low Exposure (Reference)	1.00	1.00	1.00	1.00
High Exposure	3.14 (2.94-3.35)	1.26 (1.18-1.35)	1.12 (1.02-1.24)	1.43 (1.31-1.57)
Radio				
Low Exposure (Reference)	1.00	1.00	1.00	1.00
High Exposure	2.02 (1.81-2.25)	1.29 (1.15-1.44)	1.37 (1.13-1.65)	1.22 (1.09-1.37)
Television				
Low Exposure (Reference)	1.00	1.00	1.00	1.00
High Exposure	3.92 (3.72-4.14)	1.84 (1.74-1.95)	1.39 (1.24-1.55)	2.07 (1.94-2.2)
Movie				
Less than once a month (Reference)	1.00	1.00	1.00	1.00
At least once a month	2.53 (2.34-2.74)	1.29 (1.19-1.4)	1.23 (1.09-1.38)	1.33 (1.2-1.47)

*aORs are mutually adjusted for other media exposure variables as well as age, educational attainment, current marital status, wealth, caste, and religion. OR= Odds ratio, CI= Confidence interval

For peer review only - http://bmjopen.bmj.com/site/about/guidelines.xhtml

Discussion

Media are one of the most valuable communication tools in public health. They have been, time and again, used to create awareness during public health emergencies. Mass media are a powerful tool when the target population is large and spread out, and in-person communication channels are unable to penetrate, especially through underserved rural areas.[25] Various forms of mass media have effectively educated and persuaded audiences to adopt healthy behaviors. Mass media have also been crucial in creating awareness about health-related programs and campaigns to promote healthy living and improve quality of life.[25]

Mass media exposure in South Asian countries is positively associated with utilization of maternal healthcare services.[26] Through our study, we were able to determine the associations between exposure to different types of mass media and ANC visits among Indian women during pregnancy. The descriptive characteristics established the popularity of different forms of mass media amongst Indian women. Overall, television appeared to be the most popular media source compared to newspapers/magazines, radio and watching movies in the cinema halls. According to Broadcast Audience Research Council India's recent survey, television is the preferred media of choice in India because of its affordability and its accessibility to free-to-air channels.[27] Furthermore, stratification by urbanicity suggests that high television exposure is more prevalent in urban areas, compared to rural areas. This could be because urban areas in India have better access to facilities like electricity and cable. Other contributing factors could be higher literacy rates in urban areas due to better accessibility of schools and universities.[27,28]

In both rural and urban populations, high exposure to all types of mass media was positively associated with making at least eight ANC visits (Figure 1). These findings are in accord with studies from other developing countries such as Nepal, Bangladesh, and Uganda, where they also found positive associations between mass media exposure and ANC visits.[29,30] In Malawi, community-driven mass media campaigns in the rural areas resulted in increased uptake of maternal health services including ANC, which suggests

For peer review only - http://bmjopen.bmj.com/site/about/guidelines.xhtml

that mass media can be an effective mode of intervention in limited-resource settings.[31] Interestingly, we observed that although the exposure to television and other media was higher in the urban areas, the associations were stronger in the rural areas for all types of media, except for radio.

Our study shows that high exposure to newspaper/magazine is not very prevalent amongst Indian women, especially in rural areas. However, it is positively associated with making at least eight ANC visits, in both rural as well as urban regions, after adjusting for several SES indicators. Similarly, a large percentage of both rural and urban women do not have high exposure to radio or go to the cinema to watch movies every month. However, the associations of both types of media exposure with making at least eight ANC visits was positive in both rural and urban areas. These findings imply that using newspapers/magazines, radio, or movies at cinema halls as communication tools to create awareness about utilizing adequate ANC, might have a positive impact, however, only on a smaller fraction of women. Alternatively, our data indicate that television is the most popular media source amongst women, in rural as well as urban areas. The positive association between television exposure and making at least eight ANC visits suggests that television could be an excellent media source to generate ANC awareness in women, especially in rural areas (Figure 2). Previous studies have reported that when television is used as a medium to create knowledge, knowledge gaps resulting from the communication flow of messages are less likely to occur.[32] Our study suggests that using television as a medium to create ANC awareness can have a similar effect on reducing differences in ANC behavior.

Evidently, television is the most effective media source to create awareness in our large target population. Nonetheless, even though only a small percentage of women, in both rural and urban areas, have high exposure to other media sources, it is recommended to use a multimedia approach because, in big countries like India, even a small population percentage is significant.

For peer review only - http://bmjopen.bmj.com/site/about/guidelines.xhtml

Strengths and Limitations

A major strength of this study, based on nationally representative survey data, is its generalizability. Furthermore, these data can determine trends stratified by urbanicity, states, SES, caste, and religion, which can further help to narrow down the at-risk populations and design customized interventions. Insights from this study can inform health awareness programs in other developing nations with strong mass media traditions.

One of the main limitations of this study is using self-reported NFHS-4 data, making it prone to recall bias and under- or over-reporting of behaviors. Additionally, face-to-face administration of the survey by an interviewer increases the potential for interviewer bias. The cross-sectional design of the data limits the ability to derive any causal inferences on the relationships between the variables. Nonetheless, the strong association between media exposure and ANC visits suggest the potential promise of media in maternal health promotion.

In our study, we determined the associations between media exposure and ANC visits controlling for socioeconomic factors such as women's age, education, wealth, current marital status, and religion. However, there is a possibility that other factors such as access and resource related factors, women's autonomy factors (for example, decision-making power and employment status), and partner's socio-economic factors (for example, education and employment) could also affect the associations between media exposure and making at least eight ANC visits.[26] Future research should examine how the associations between media exposure and ANC visits in India are modified by these additional factors. It is also important to note that, in this study, we only determined the association between the four most common mass media sources and making at least eight ANC visits. However, other modes are also used to create ANC-related awareness in India, such as social media, billboards, cellphones, community theater, medical camps, door-to-door information by community healthcare workers, and pamphlet distributions.[23,33] Moreover, other sources of media such as the internet, computers, and smartphones are gaining popularity in India. Future surveys should also include

For peer review only - http://bmjopen.bmj.com/site/about/guidelines.xhtml

information regarding their use and their implications on health-related behaviors. NFHS-4 has no information about the type of ANC-related media content available to women in India. Thus, one can only speculate based on literature. Future studies should examine ANC-related media content to obtain a complete picture of the kind of information encouraging such behaviors.

Conclusion

In India, there is a need for increased awareness about obtaining adequate ANC visits. Positive associations between high exposure to different forms of mass media and making at least eight ANC visits provide evidence that exposure to media may encourage positive pregnancy behaviors. In both rural and urban India, the popularity of television and its association with making at least eight ANC visits is stronger than other forms of media. ANC related health awareness campaigns in the country can use these findings to allocate resources to the appropriate forms of mass media to encourage healthy behaviors. Moreover, clearly, using mass media, especially television, can promote maternal health at a scale, unlike other more labor-intensive approaches.

Author Contributions DD and KV conceived and designed the study. RP and MB contributed to the design of the study. DD analyzed and interpreted the data and wrote the first draft of the manuscript. DD, RP, MB and KV contributed to the interpretation of the data and revised the manuscript for important intellectual content. All authors provided critical feedback on the manuscript and agree to be accountable for all aspects of the work.

Funding This research was supported by funding from the Bill & Melinda Gates Foundation (Viswanath, PI), grant number INV-005090. The funders had no role in the study design; in the collection, analysis and interpretation of the data; in the writing of the report; and in the decision to submit the paper for publication.

Competing Interests None declared

Patient consent for publication Not required.

For peer review only - http://bmjopen.bmj.com/site/about/guidelines.xhtml

Ethics approval The Institutional Review Board of Tufts University School of Medicine/Tufts Medical Center waived the need for ethics approval because this is a cross-sectional study using the publicly available NFHS-4 dataset, which did not constitute human subjects research, did not collect identifying information and was intended for quality improvement/quality assurance purposes only.

Provenance and peer review Not commissioned; externally peer reviewed.

Data availability statement Data are available in a public, from the Demographic and Health Surveys Program.

For peer review only

For peer review only - http://bmjopen.bmj.com/site/about/guidelines.xhtml

Bibliography

1 World Health Organization. *WHO Recommendation on Antenatal care for positive pregnancy experience*. 2016. doi:ISBN 978 92 4 154991 2

2 Kumar G, Choudhary TS, Srivastava A, *et al.* Utilisation, equity and determinants of full antenatal care in India: analysis from the National Family Health Survey 4. *BMC Pregnancy Childbirth* 2019;**19**:327. doi:10.1186/s12884-019-2473-6

3 Dixit P, Dwivedi LK, Ram F. Strategies to Improve Child Immunization via Antenatal Care Visits in India: A Propensity Score Matching Analysis. *PLoS One* 2013;**8**:e66175. doi:10.1371/journal.pone.0066175

4 World Health Organization. New guidelines on antenatal care for a positive pregnancy experience. World Health Organization 2017. https://www.who.int/reproductivehealth/news/antenatal-care/en/ (accessed 1 Apr 2020).

5 Doku DT, Neupane S. Survival analysis of the association between antenatal care attendance and neonatal mortality in 57 low- And middle-income countries. *Int J Epidemiol* 2017;**46**:1668–77. doi:10.1093/ije/dyx125

6 UNICEF. Antenatal Care - UNICEF data. 2019. https://data.unicef.org/topic/maternal-health/antenatal-care/# (accessed 5 May 2020).

7 Viswanath K, Ramanadhan S, Kontos EZ. Mass Media. In: *Macrosocial Determinants of Population Health*. New York, NY: : Springer New York 2007. 275–94. doi:10.1007/978-0-387-70812-6_13

8 Hornik R, Yanovitzky I. Using theory to design evaluations of communication campaigns: The case of the national youth anti-drug media campaign. *Commun Theory* 2003;**13**:204–24. doi:10.1111/j.1468-2885.2003.tb00289.x

9 Wakefield MA, Loken B, Hornik RC. Use of mass media campaigns to change health behaviour. *Lancet* 2010;**376**:1261–71. doi:10.1016/S0140-6736(10)60809-4

10 Fishbein M, Ajzen I. Predicting and changing behavior: The reasoned action approach. *Predict Chang Behav Reason Action Approach* 2011;:1–518. doi:10.4324/9780203838020

11 Video Gallery | PMSMA. https://pmsma.nhp.gov.in/media-gallery/video-gallery/ (accessed 29 Oct 2020).

12 Singh PK. India has achieved groundbreaking success in reducing maternal mortality. WHO Reg. Off. South-East Asia. 2018.https://www.who.int/southeastasia/news/detail/10-06-2018-india-has-achieved-groundbreaking-success-in-reducing-maternal-mortality (accessed 29 Oct 2020).

13 Jones G, Steketee RW, Black RE, *et al.* How many child deaths can we prevent this year? *Lancet* 2003;**362**:65–71. doi:10.1016/S0140-6736(03)13811-1

14 Kuhnt J, Vollmer S. Antenatal care services and its implications for vital and

For peer review only

health outcomes of children: Evidence from 193 surveys in 69 low-income and middle-income countries. *BMJ Open* 2017;**7**. doi:10.1136/bmjopen-2017-017122

15 National Family Health Survey. National Family Health Survey , India. 2017. http://rchiips.org/nfhs/NFHS-4Reports/India.pdf (accessed 10 May 2020).

16 Ghosh R. Child mortality in India: A complex situation. *World J Pediatr* 2012;**8**:11–8. doi:10.1007/s12519-012-0331-y

17 World Health Organization G, Fund UNC, Fund UNP, *et al. Trends in maternal mortality 2000 to 2017: estimates by WHO, UNICEF, UNFPA, World Bank Group and the United Nations Population Division.* 2019.

18 UNICEF. *Children , food and nutrition: growing well in a changing world*. 2019.

19 Janani Suraksha Yojana :: National Health Mission. https://nhm.gov.in/index1.php?lang=1&level=3&sublinkid=841&lid=309 (accessed 27 Apr 2020).

20 Lim SS, Dandona L, Hoisington JA, *et al.* India's Janani Suraksha Yojana, a conditional cash transfer programme to increase births in health facilities: an impact evaluation. *Lancet* 2010;**375**:2009–23. doi:10.1016/S0140-6736(10)60744-1

21 Murukutla N, Yan H, Wang S, *et al.* Cost-effectiveness of a smokeless tobacco control mass media campaign in India. *Tob Control* 2018;**27**:547–51. doi:10.1136/tobaccocontrol-2016-053564

22 Curtis V. Explaining the outcomes of the 'Clean India' campaign: Institutional behaviour and sanitation transformation in India. *BMJ Glob Heal* 2019;**4**:e001892. doi:10.1136/bmjgh-2019-001892

23 JAN ANDOLAN-Guidelines. https://icds-wcd.nic.in/nnm/NNM-Web-Contents/LEFT-MENU/Guidelines/JanAndolanGuidelines-English.pdf (accessed 5 May 2020).

24 Saroha E, Altarac M, Sibley LM. Caste and Maternal Health Care Service Use Among Rural Hindu Women in Maitha, Uttar Pradesh, India. *J Midwifery Women's Heal* 2008;**53**:41–7. doi:10.1016/j.jmwh.2008.05.002

25 unite for sight. Health Communication Course: Module 5 - The Role of Media in Health Promotion. Unite for Sight. 2010.https://www.uniteforsight.org/health-communication-course/module5 (accessed 3 Dec 2019).

26 Fatema K, Lariscy JT. Mass media exposure and maternal healthcare utilization in South Asia. *SSM - Popul Heal* 2020;**11**. doi:10.1016/j.ssmph.2020.100614

27 BARC India. The Changing Faces of TV in India. https://www.barcindia.co.in/resources/The Changing Face of TV in India.pdf (accessed 7 May 2020).

28 Implementation P, Office NS. Key Indicators of Household Social Consumption on Education in India Government of India Ministry of Statistics and Programme Implementationin GoIStats GoIStats goistats. 2017. http://www.mospi.gov.in/sites/default/files/publication_reports/KI_Education_75th_

For peer review only - http://bmjopen.bmj.com/site/about/guidelines.xhtml

Final.pdf (accessed 8 Dec 2019).

29 Acharya D, Khanal V, Singh JK, *et al.* Impact of mass media on the utilization of antenatal care services among women of rural community in Nepal. *BMC Res Notes* 2015;**8**:345. doi:10.1186/s13104-015-1312-8

30 Bbaale E. Factors influencing the utilisation of antenatal care content in Uganda. *Australas Med J* 2011;**4**:516–26. doi:10.4066/AMJ.2011.849

31 Zamawe COF, Banda M, Dube AN. The impact of a community driven mass media campaign on the utilisation of maternal health care services in rural Malawi. *BMC Pregnancy Childbirth* 2016;**16**:21. doi:10.1186/s12884-016-0816-0

32 Viswanath K, Finnegan JR. The Knowledge Gap Hypothesis: Twenty-Five Years Later. In: *Annals of the International Communication Association*. 1996. 187–228. doi:10.1080/23808985.1996.11678931

33 Chandra A, Sarkar S, Adinarayanan S, *et al.* Impact of health awareness campaign in improving the perception of the community about palliative care: A pre- and post-intervention study in rural Tamil Nadu. *Indian J Palliat Care* 2016;**22**:467–76. doi:10.4103/0973-1075.191832

Figure Legends

Figure 1: Association between high exposure to different types of mass media and making at least eight antenatal care visits, stratified by urbanicity. (Error bars = 95% confidence intervals)

Figure 2: Association between high exposure to television and making at least eight antenatal care visits, stratified by urbanicity. (Error bars = 95% confidence intervals)

For peer review only

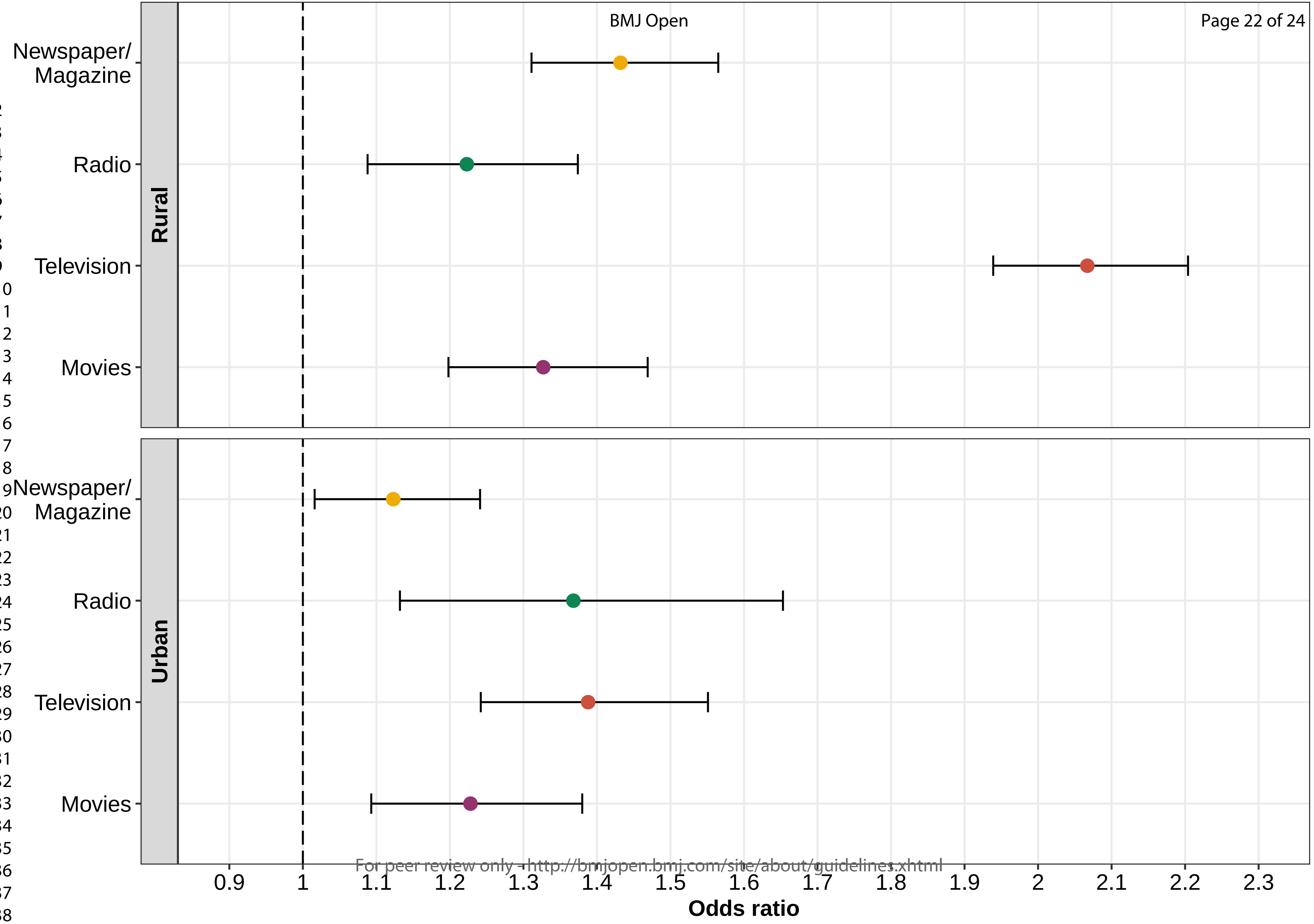
Rural
Urban
Newspaper/ Magazine
Radio
Television
Movies
0.9
1
1.1
1.2
1.3
1.4
1.5
1.6
1.7
1.8
1.9
2
2.1
2.2
2.3
Odds ratio

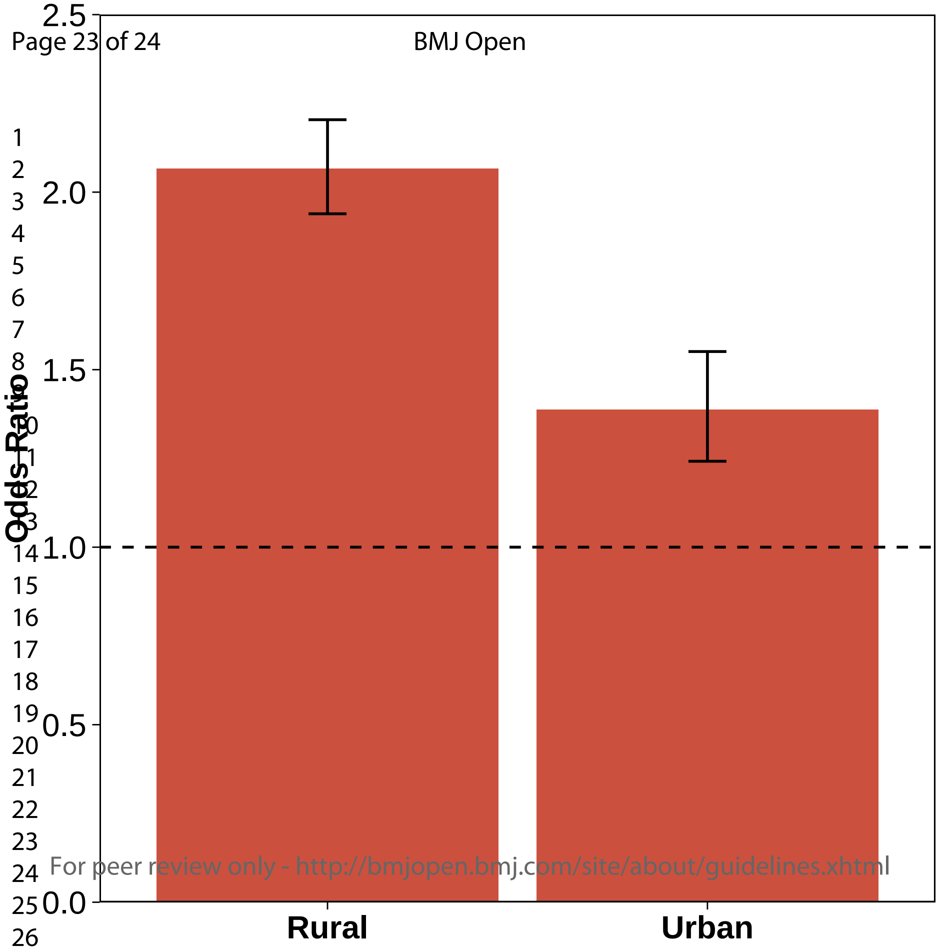
2.5
2.0
1.5
1.0
0.5
0.0
Odds Ratio
Rural
Urban

STROBE 2007 (v4) Statement—Checklist of items that should be included in reports of *cross-sectional studies*

Section/Topic	Item #	Recommendation	Reported on page #
**Title and abstract**	1	(*a*) Indicate the study's design with a commonly used term in the title or the abstract	2
		(*b*) Provide in the abstract an informative and balanced summary of what was done and what was found	2
**Introduction**			
Background/rationale	2	Explain the scientific background and rationale for the investigation being reported	6
Objectives	3	State specific objectives, including any prespecified hypotheses	6
**Methods**			
Study design	4	Present key elements of study design early in the paper	7
Setting	5	Describe the setting, locations, and relevant dates, including periods of recruitment, exposure, follow-up, and data collection	7
Participants	6	(*a*) Give the eligibility criteria, and the sources and methods of selection of participants	7
Variables	7	Clearly define all outcomes, exposures, predictors, potential confounders, and effect modifiers. Give diagnostic criteria, if applicable	7,8
Data sources/ measurement	8*	For each variable of interest, give sources of data and details of methods of assessment (measurement). Describe comparability of assessment methods if there is more than one group	7,8
Bias	9	Describe any efforts to address potential sources of bias	7
Study size	10	Explain how the study size was arrived at	7
Quantitative variables	11	Explain how quantitative variables were handled in the analyses. If applicable, describe which groupings were chosen and why	7,8
Statistical methods	12	(*a*) Describe all statistical methods, including those used to control for confounding	9
		(*b*) Describe any methods used to examine subgroups and interactions	9
		(*c*) Explain how missing data were addressed	7
		(*d*) If applicable, describe analytical methods taking account of sampling strategy	n/a
		(*e*) Describe any sensitivity analyses	n/a
**Results**			

For peer review only

Participants	13*	(a) Report numbers of individuals at each stage of study—eg numbers potentially eligible, examined for eligibility, confirmed eligible, included in the study, completing follow-up, and analysed	7
		(b) Give reasons for non-participation at each stage	7
		(c) Consider use of a flow diagram	n/a
Descriptive data	14*	(a) Give characteristics of study participants (eg demographic, clinical, social) and information on exposures and potential confounders	9
		(b) Indicate number of participants with missing data for each variable of interest	6
Outcome data	15*	Report numbers of outcome events or summary measures	9
Main results	16	(*a*) Give unadjusted estimates and, if applicable, confounder-adjusted estimates and their precision (eg, 95% confidence interval). Make clear which confounders were adjusted for and why they were included	10
		(*b*) Report category boundaries when continuous variables were categorized	n/a
		(*c*) If relevant, consider translating estimates of relative risk into absolute risk for a meaningful time period	n/a
Other analyses	17	Report other analyses done—eg analyses of subgroups and interactions, and sensitivity analyses	10
**Discussion**			
Key results	18	Summarise key results with reference to study objectives	13
Limitations	19	Discuss limitations of the study, taking into account sources of potential bias or imprecision. Discuss both direction and magnitude of any potential bias	15
Interpretation	20	Give a cautious overall interpretation of results considering objectives, limitations, multiplicity of analyses, results from similar studies, and other relevant evidence	13,14
Generalisability	21	Discuss the generalisability (external validity) of the study results	14,15
**Other information**			
Funding	22	Give the source of funding and the role of the funders for the present study and, if applicable, for the original study on which the present article is based	16

*Give information separately for cases and controls in case-control studies and, if applicable, for exposed and unexposed groups in cohort and cross-sectional studies.

Note: An Explanation and Elaboration article discusses each checklist item and gives methodological background and published examples of transparent reporting. The STROBE checklist is best used in conjunction with this article (freely available on the Web sites of PLoS Medicine at http://www.plosmedicine.org/, Annals of Internal Medicine at http://www.annals.org/, and Epidemiology at http://www.epidem.com/). Information on the STROBE Initiative is available at www.strobe-statement.org.

For peer review only
